# Patterns, timing, and predictors of recurrence after laparoscopic liver resection for hepatocellular carcinoma: results from a high-volume HPB center

**DOI:** 10.1007/s00464-021-08390-5

**Published:** 2021-02-23

**Authors:** Haili Zhang, Fei Liu, Ningyuan Wen, Bo Li, Yonggang Wei

**Affiliations:** 1 Department of Liver Surgery & Liver Transplantation Center, West China Hospital of Sichuan University, 37 Guo Xue Road, Wuhou District, Chengdu, 610041 China; 2 West China School of Medicine, Sichuan University, Chengdu, 610041 China

**Keywords:** Hepatocellular carcinoma, Recurrence patterns, Laparoscopic liver resection, Recurrence-free survival

## Abstract

**Background:**

Although long-term outcomes may be comparable between laparoscopic liver resection (LLR) and open liver resection (OLR) for hepatocellular carcinoma (HCC), there has been little discussion regarding the patterns of recurrence after LLR.

**Methods:**

Patients with HCC who underwent hepatectomy between April 2015 and November 2018 were included in this study. The recurrence patterns were analyzed in detail. The recurrence outcomes following laparoscopic versus OLR for HCC were compared after 1:2 propensity score matching. Potential risk factors for recurrence were also assessed with Cox proportional risk models.

**Results:**

Among 425 patients after LLR, 144 (33.8%) experienced recurrence at the last follow-up, with a median recurrence-free survival (RFS) of 10.0 months (range 1–58 months). The most frequent recurrence site was the liver (*n* = 99, 68.8%), followed by the surgical margin (*n* = 15, 10.4%) and distant metastases (*n* = 12, 8.3%). Liver recurrence with distant metastasis (*n* = 10, 6.9%) tended to occur early (median 8.0 months), while peritoneal recurrence (*n* = 8, 5.6%) occurred later (median 14.0 months). A total of 120 (83.3%) patients had recurrence within 2 years after LLR. No trocar site recurrence was observed in this study. The recurrence patterns, timing, and treatment did not show significant differences between the LLR and OLR. The independent risk factors for recurrence included ALBI grade, postoperative α-fetoprotein > 8 ng/ml, tumor size > 5 cm, surgical margin ≤ 1 cm, and multiple tumors. Patients with recurrence had 1- and 5-year overall survival rates of 81.1% and 60.7%, respectively, compared with rates of 95.8% and 92.9% for patients without recurrence (*P* < 0.000).

**Conclusion:**

This study suggested that intrahepatic recurrence was still the most common recurrence pattern for HCC after LLR and that LLR did not increase the risk of trocar hole recurrence or implantation. Most cases of recurrence occurred within 2 years after LLR, suggesting that surveillance should be targeted to early recurrence.

**Supplementary Information:**

The online version of this article (10.1007/s00464-021-08390-5) contains supplementary material, which is available to authorized users.

Liver cancer remains an important cancer worldwide, and it was responsible for over 841,000 new cases and an estimated 782,000 deaths in 2018, making it the sixth most commonly diagnosed cancer and the fourth leading cause of cancer-related death [1]. Although major advances in surgical technology and surveillance have allowed a better prognosis for HCC patients, 50–70% of patients experience tumor recurrence within 5 years after surgery [2, 3].

Recently, advances in minimally invasive techniques and perioperative management have resulted in a higher proportion of patients eligible for laparoscopic liver resection (LLR). Minimally invasive surgery is becoming mainstream, as laparoscopic surgery has been reported to have satisfactory long-term outcomes compared with open surgery according to meta-analyses and large propensity score-matched studies [4, 5]. LLR for HCC is associated with reduced negative effects on both postoperative and oncological outcomes, including blood loss, liver failure, and complications [6–8]. Simultaneously, cases that require complex resection, such as tumors located in unfavorable segments, large tumors, and tumors in close proximity to major vessels, also require external landmarks, intraoperative ultrasound, and selective clamping using Glissonian access [9], which represent difficult challenges. As a result, LLR has different intrinsic properties from open liver resection (OLR), and its long-term outcomes need to be separately analyzed. Although many studies have focused on HCC recurrence in conventional open hepatectomy [3, 10, 11], relevant discussions are currently lacking in LLR. Therefore, the aim of this study was to present the patterns and timing of recurrence after LLR for HCC using data from a high-volume HPB center. Moreover, the risk factors predicting recurrence for HCC after LLR were also studied.

## Methods

Data from all HCC patients (HCC was diagnosed based on histologic analyses) who underwent liver resection at West China Hospital of Sichuan University were retrieved from the registry and follow-up databases. The exclusion criteria were mixed liver cancer; histologically positive surgical margins; a history of cancer; and preoperative therapy such as hepatectomy, transarterial chemoembolization (TACE), and radiofrequency ablation (RFA). This study was approved by the institutional review board.

Resectability and staging were estimated using abdominal computed tomography (CT) and were typically discussed by an expert team. Our detailed techniques for LLR have been previously described [12–14]. Briefly, the operation was performed under general anesthesia, and carbon dioxide was infused to maintain a pneumoperitoneum pressure of 12 ~ 14 mmHg; patients were placed in the semi-left lateral position and reversed Trendelenburg position. Hepatic inflow occlusion methods, the intermittent Pringle maneuver, or continuous hemi-hepatic vascular inflow occlusion was used to control surgical blood loss. Parenchymal transection of the liver was performed with a Harmonic scalpel, a Cavitron ultrasonic surgical aspirator (CUSA), or Ligasure [15] with the central venous pressure maintained at < 5 mmHg. For OLR, all patients under the same anesthesia protocol were placed in the supine position, and laparotomy was performed through a reverse L-incision. A Harmonic scalpel, a CUSA, or Ligasure was used as the main method for parenchyma transection. Pringle maneuvers and bipolar electrocoagulation procedures were usually applied to control blood loss. In both operation procedures, intraoperative ultrasonography was performed routinely to identify the location, size, and number of tumors; to identify the adjacent vasculature; and to maintain an appropriate resection margin.

All specimens were histologically assessed by two experienced pathologists. Information about the resection margin, tumor differentiation, the tumor number, microvascular invasion, major vascular invasion, bile duct invasion, satellite nodules, and Ishak scores was recorded.

### Perioperative work-up and follow-up

All patients underwent routine blood tests before and after surgery, and abdominal ultrasonography was performed before discharge from the hospital. The preoperative albumin-bilirubin (ALBI) grade was calculated from available data as a measure of liver reserve function [equation: score = (log_10_ bilirubin µmol/L * 0.66) + (albumin g/L * − 0.085); the ALBI scores were further classified into three different grades: grade 1 (less than − 2.60), grade 2 (between − 2.60 and − 1.39), and grade 3 (above − 1.39)] [16]. AFP values were collected within 1 week before and 3 months after surgery. The diagnosis of liver cirrhosis was based on histological examination. With the Ishak staging system, a score of 5 or 6 points is defined as liver cirrhosis [17].

Patients were followed in the outpatient department and by telephone regularly. In general, patients were requested to complete follow-up blood tests (liver function and tumor markers) and abdominal ultrasonography every 3 months. At least one enhanced computed tomography (CT) and/or magnetic resonance imaging (MRI) scan was carried out every 6 months. Gadolinium-ethoxy-benzyl-diethylenetriamine pentaacetic acid (Gd-EOB-DTPA)-enhanced MRI was performed when necessary. All diagnoses of recurrent HCC were based on positive imaging findings, including enhanced CT and/or MRI [2, 18]. If the patient had relevant laboratory abnormalities and symptoms, we further arranged imaging examinations for recurrence screening. Information about recurrence patterns was extracted from imaging. The recurrence location (surgical margin or not), number, size, and treatment of recurrent tumors in the liver were recorded. When considering patterns of recurrence, only the first recurrence was documented in this study. The decision regarding recurrence therapy was made based on performance status and recurrence patterns. Generally, recurrence therapies included liver resection, RAF, TACE, and liver transplantation.

### Statistical analysis

Continuous variables were compared using *t* tests or Mann–Whitney *U* tests (when the variables did not coincide with a normal distribution) and are described as the median (interquartile range, IQR). Categorical variables were compared using *χ*^2^ tests or Fisher’s exact *t*ests and are expressed as percentages. Age, BCLC stage, and tumor size were selected as covariates, and 1:2 matching between the LLR and OLR groups was performed within a caliper value of 0.02. Overall survival (OS) and recurrence-free survival (RFS) were calculated using the Kaplan–Meier method and compared using the log-rank test. Potentially meaningful variables identified by univariate analysis were selected for multivariate analysis of Cox proportional risk models to determine the independent risk factors associated with recurrence. Significance levels were set at 0.05, and all analyses were 2 tailed. Statistical analyses were performed using SPSS software 22.0 (IBM SPSS Inc., Chicago, IL) and R 3.3.1 (https://cran.r-project.org/).

## Results

### Baseline characteristics

A total of 437 patients who underwent LLR for primary HCC in our center between April 2015 and November 2018 were included. Among them, 1 patient had HCC rupture, and 11 patients had missing clinical data. Finally, 425 patients were enrolled in the study. The demographic, clinicopathologic, and treatment characteristics of all patients, as well as the differences between patients with and without recurrence, are summarized in Table 1. There were 902 patients in the OLR group; after 1:2 PSM, 398 patients in the LLR group and 599 patients in the OLR group were analyzed (not all LLR units received 2 matches). The baseline characteristics and clinicopathologic and short-term outcomes before and after PSM are summarized in Supplementary Table 1. The distributions of propensity scores before and after matching are shown in Supplementary Fig. 1.

**Table 1:** Demographics, clinicopathologic, and treatment characteristics of included patients

Variable	All patients (*n* = 425)	No recurrence (*n* = 281)	Recurrence (*n* = 144)	*P* value
Age, mean years (SD)	53.8 (11.3)	54.3 (11.8)	53.1 (10.5)	0.115
Male, *n* (%)	357 (84.0)	237 (84.3)	120 (83.3)	0.788
BMI > 27, *n* (%)	54 (12.7)	34 (12.1)	20 (13.9)	0.610
ALBI grade, *n* (%)				0.235
1	344 (80.9)	232 (82.6)	112 (77.8)	
2	81 (19.1)	49 (17.4)	32 (22.2)	
BCLC stage, *n* (%)				0.010
A	368 (86.6)	253 (90.0)	115 (79.9)	
B	47 (11.0)	22 (7.8)	25 (17.4)	
C	10 (2.4)	6 (2.1)	4 (2.8)	
Etiology				0.736
HBV	376 (88.5)	247 (87.9)	129 (89.6)	
HCV	2 (0.5)	1 (0.4)	1 (0.7)	
Other	47 (11.1)	33 (11.7)	14 (9.7)	
HBV-DNA + , *n* (%)	245 (57.8)	156 (55.7)	89 (61.8)	0.229
Pre-AFP (ng/ml), median (IQR)	54.4 (4.8–885.0)	23.1 (4.0–511.7)	125.8 (10.7–1210)	0.012
Post-AFP (ng/ml), median (IQR)	5.6 (3.0–32.6)	4.2 (2.7–14.3)	13.3 (4.5–97.3)	0.000
Portal hypertension, *n* (%)	84 (19.8)	46 (6.4)	38 (6.4)	0.014
Operation procedure, *n* (%)				0.027
Anatomical resection	237 (55.8)	146 (52.0)	91 (63.2)	
Non-anatomical resection	188 (44.2)	135 (48.0)	53 (36.8)	
Difficulty score, median (IQR)	6 (4–7)	5 (4–7)	6 (4–8)	0.032
Operation time > 3 h, *n* (%)	258 (60.7)	163 (58.0)	95 (66.0)	0.112
Pringle, *n* (%)	333 (78.4)	222 (79.0)	111 (77.1)	0.544
CHVIO, *n* (%)	19 (4.5)	14 (5.0)	5 (3.5)	0.659
Complications, *n* (%)				0.914
Clavien-Dindo grade ≤ II	405 (95.3)	268 (95.4)	137 (95.1)	
Clavien-Dindo grade ≥ III	20 (4.7)	13 (4.6)	7 (4.9)	
Resection margin, *n* (%)				0.049
> 1 cm	120 (28.2)	88 (31.3)	32 (22.2)	
≤ 1 cm	305 (71.8)	193 (68.7)	112 (77.8)	
Tumor location, *n* (%)				0.027
Right/left liver	16 (3.8)	11 (7.6)	5 (1.8)	
Right anterior section	17 (4.0)	4 (2.8)	13 (4.6)	
Right posterior section	20 (4.7)	7 (4.9)	13 (4.6)	
Posterosuperior segment	96 (22.6)	27 (18.8)	69 (24.6)	
Anterolateral segment	276 (64.9)	95 (66.0)	181 (64.4)	
Blood loss (ml), median (IQR)	200 (70–400)	150 (50–300)	200 (100–400)	0.025
Tumor size, median cm (IQR)	3.5 (2.5–5.0)	3.5 (2.2–4.8)	4.4 (3.0–6.0)	0.000
Tumor number, *n* (%)				0.003
Single	354 (83.3)	245 (87.2)	109 (75.7)	
Multiple	71 (16.7)	36 (12.8)	35 (24.3)	
Tumor differentiation, *n* (%)				0.046
Well moderate	264 (61.1)	184 (65.5)	80 (55.6)	
Poor	161 (38.9)	97 (34.5)	64 (44.4)	
Microvascular invasion, *n* (%)	87 (20.5)	53 (18.9)	34 (23.6)	0.251
Portal vein invasion, *n* (%)	9 (2.1)	5 (1.8)	4 (2.8)	0.499
Satellite nodules, *n* (%)	21 (4.9)	9 (3.2)	12 (8.3)	0.021
Cirrhosis, *n* (%)	260 (61.2)	165 (58.7)	95 (66.0)	0.146

*BMI* body mass index, *ALBI* albumin-bilirubin, *BCLC* barcelona clinic liver cancer, *HBV* hepatitis B virus, *HCV* hepatitis C virus, *Pre-AFP* preoperative α-fetoprotein, *Post-AFP* postoperative α-fetoprotein, *CHVIO* continuous hemi-hepatic vascular inflow occlusion, *SD* standard deviation, *IQR* interquartile range;

**Fig. 1:**
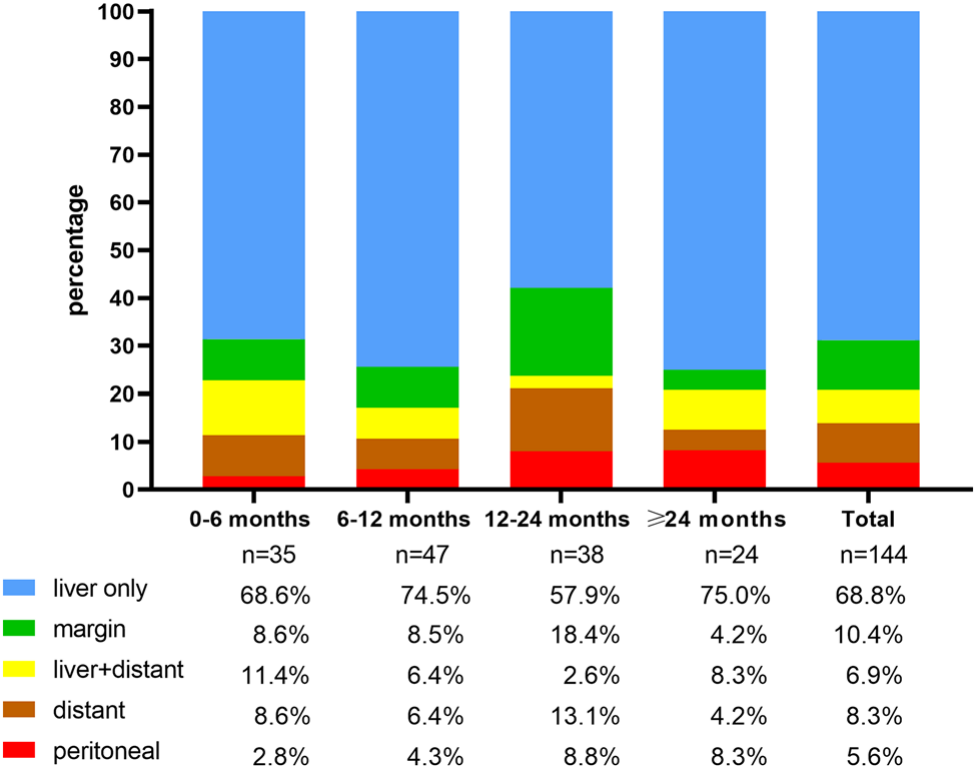
Distribution of recurrence patterns at different time points

### Patterns and timing of recurrence after LLR

**Fig. 2:**
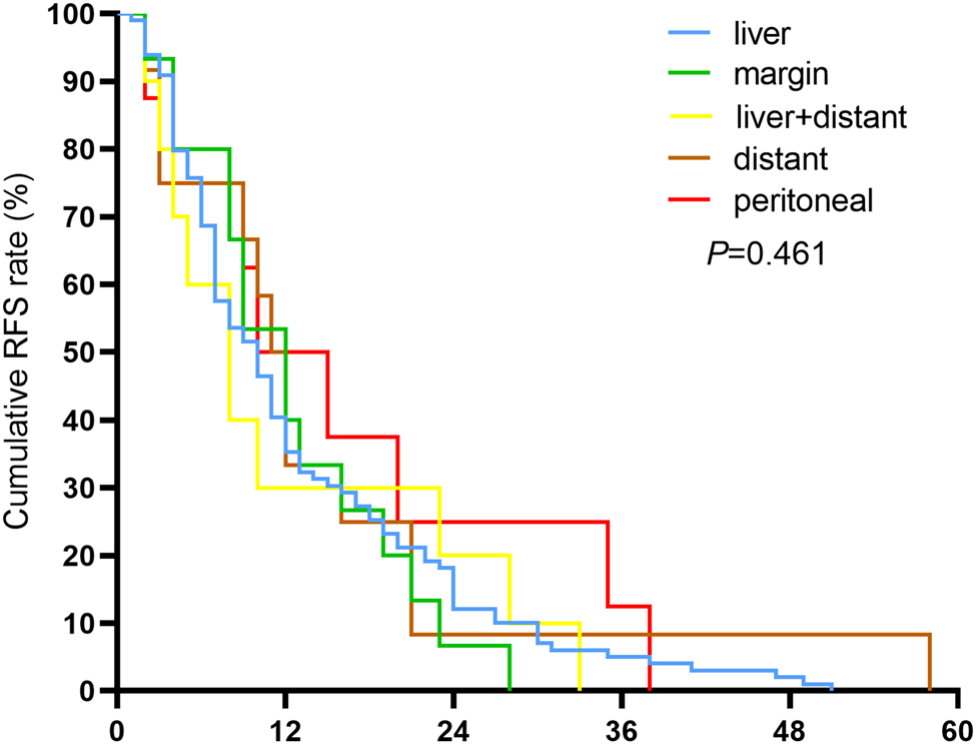
Recurrence-free survival curves for different recurrence patterns

The median follow-up time for the entire cohort was 26.0 months (range 1–61). Among the 425 included patients, 144 (33.8%) had HCC recurrence at the last follow-up, with a median RFS time of 10.0 months (range 1–58). In terms of the recurrence sites (Fig. 1), 99 (68.8%) patients were diagnosed with liver-only recurrence, with a median RFS of 10.0 months (range 1–51), and 15 (10.4%) patients experienced surgical margin recurrence, with a median RFS of 12 months (range 2–28). Ten (6.9%) patients had liver recurrence with distant metastasis, with a median RFS of 8 months (range 3–23), and 8 (5.6%) patients had peritoneal-only recurrence, with a median RFS of 14 months (range 2–38). RFS values did not differ significantly from different patterns (*P* = 0.461, Fig. 2). Another 12 (8.3%) patients had distant metastasis (median RFS 12 months, range 3–58 months) found in the lung, osseous structures, brain, and lymph nodes. Among 114 (79.2%) patients with recurrent liver lesions (liver-only and margin recurrence), only 45 (39.8%) had single lesions, while the median tumor size was 2.0 cm (range 0.6–11.2 cm). No patients experienced recurrence/metastasis at the specimen extraction incision sites or trocar holes.

Among the 144 patients who experienced recurrence, 120 (83.3%) experienced recurrence within 24 months, and 24 (16.7%) experienced recurrence beyond 24 months after the operation. The recurrence patterns did not change over time, and intrahepatic recurrence was still the most common recurrence pattern for HCC after LLR (Fig. 1).

### Treatment of recurrence

**Fig. 3:**
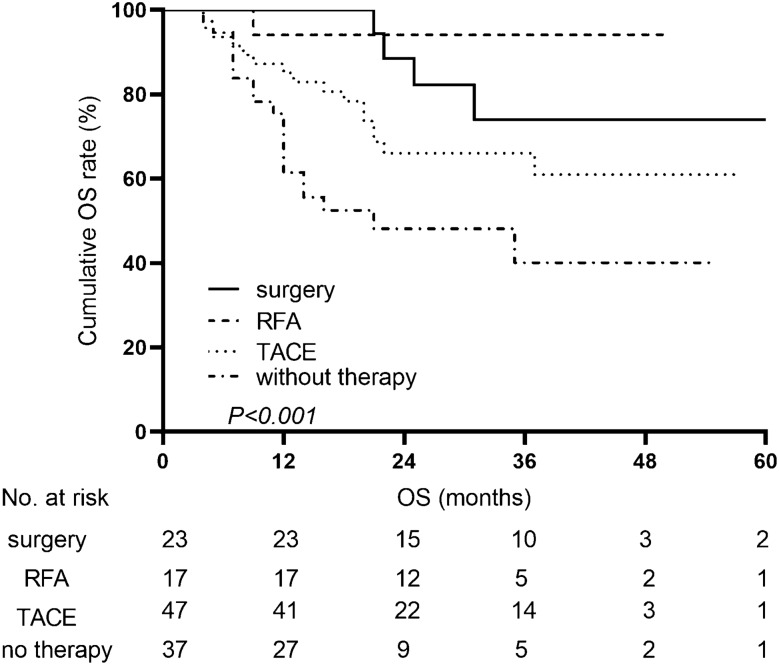
Overall survival curves for different recurrence therapies

In the recurrence group, 23 (16.0%) patients were treated with repeat hepatectomy, 17 (11.8%) patients were treated with RFA, 47 (32.6%) patients were treated with TACE, and only 1 (0.7%) patient received liver transplantation. The OS values between patients who underwent surgery and RFA were similar, but these patients had better survival than patients who only received TACE for the treatment of recurrence. Recurrent patients without therapy showed the worst OS (*P* < 0.001) (Fig. 3).

### Patterns and timing of recurrence compared between LLR and OLR after PSM

A total of 139 (34.9%, LLR group) and 179 (29.9%, OLR group) patients had HCC recurrence at the last follow-up, with median RFS times of 10 months (range 1–58) and 10 months (range 1–51), respectively. There were no significant differences between the two groups regarding the recurrence patterns, treatments (Table 2), or RFS values (Supplementary Fig. 2).

**Table 2:** Recurrence patterns, timing, and treatments between LLR and OLR after PSM

Variable	LLR (*n* = 139)	OLR (*n* = 179)	*P* value
Patterns, *n* (%)			
Liver only	96 (69.1)	125 (69.8)	0.883
Margin	14 (10.1)	19 (10.6)	0.875
Liver & distant	10 (7.2)	12 (6.7)	0.864
Distant	11 (7.9)	19 (10.6)	0.414
Peritoneal	8 (5.8)	4 (2.2)	0.303
RFS, median mo. (range)	10 (1–58)	10 (1–51)	0.686
Treatment, *n* (%)			0.086
Surgery	23 (16.5)	33 (18.4)	
RFA	17 (12.2)	33 (18.4)	
TACE	46 (33.1)	71 (39.7)	
Radiotherapy	4 (2.9)	4 (2.2)	

*LLR* laparoscopic liver resection, *OLR* open liver resection, *PSM* propensity score match, *RFS* recurrence-free survival, *RFA* radiofrequency ablation, *TACE* transarterial chemoembolization

### Subgroup analyses

The patients in the LLR group were divided into subgroups according to the type of resection (anatomical resection, AR; or non-anatomical resection, NAR). There was a slightly higher margin recurrence rate in the NAR group, although there was no significant difference (15.1% vs. 7.7%, *P* = 0.161) (Table 3). The overall RFS values were comparable between the two groups (Supplementary Fig. 3).

**Table 3:** Recurrence patterns and timing between AR and NAR in LLR group

Variable	AR (*n* = 91)	NAR (*n* = 53)	*P* value
Patterns, *n* (%)			
Liver only	62 (68.1)	37 (69.8)	0.834
Margin	7 (7.7)	8 (15.1)	0.161
Liver & distant	7 (7.7)	3 (5.7)	0.644
Distant	10 (11.0)	2 (3.8)	0.131
Peritoneal	5 (5.5)	3 (5.7)	0.967
RFS, median mo. (range)	9 (1–58)	11 (2–47)	0.164

*AR* anatomical resection, *NAR* non-anatomical resection, *LLR* laparoscopic liver resection, *RFS* recurrence-free survival

Additional subgroup analyses by risk factors in multivariate analysis are summarized in Supplementary Table 2.

### Risk factors associated with HCC recurrence after LLR

The multivariate analysis indicated that ALBI grade (HR 1.69, 95% CI 1.19–2.18, *P* = 0.039), postoperative α-fetoprotein (AFP) > 8 ng/ml (HR 2.99, 95% CI 2.64–3.33, *P* < 0.001), tumor size > 5 cm (HR 2.84, 95% CI 2.46–3.21, *P* < 0.001), surgical margin ≤ 1 cm (HR 1.95, 95% CI 1.53–2.67, *P* = 0.002), and multiple tumors (HR 2.70, 95% CI 1.94–3.46, *P* = 0.010) were independently associated with HCC recurrence after curative resection (Table 4).

**Table 4:** Univariable and Multivariable risk factors for recurrence in HCC after LLR

Risk factors	Univariable		Multivariable	
	HR (95% CI)	*P* value	HR (95% CI)	*P* value
Age: > 60 years vs. ≤ 60 years	0.85 (0.59–1.21)	0.358		
Sex: male vs. female	0.95 (0.60–1.48)	0.818		
BMI: > 27 vs. ≤ 27	1.02 (0.63–1.65)	0.952		
ALBI: 2 vs. 1	**1.67 (1.09–2.40)**	**0.019**	**1.69 (1.19–2.18)**	**0.039**
BCLC stage	2.35 (1.54–3.57)	< 0.001		
Portal hypertension: yes vs. no	1.68 (1.13–2.51)	0.011		
Operation procedure: anatomical vs. non-anatomical	1.35 (0.96–1.89)	0.085		
Surgical margin: > 1 vs. ≤ 1 cm	**1.61 (1.08–2.40)**	**0.019**	**1.95 (1.53–2.37)**	**0.002**
Operation time: ≥ 3 vs. < 3 h	1.85 (0.98–2.04)	0.064		
Complications: Clavien ≥ III vs. Clavien ≤ II	1.27 (0.59–2.27)	0.535		
Pringle: yes vs. no	0.85 (0.56–1.30)	0.561		
Post-AFP: > 8 vs. ≤ 8 ng/ml	**2.94 (2.11–4.11)**	** < 0.001**	**2.98 (2.64–3.33)**	** < 0.001**
Size: ≥ 5 vs. < 5 cm	**2.73 (1.96–3.79)**	** < 0.001**	**2.83 (2.46–3.21)**	** < 0.001**
Number: multiple vs. single	**2.15 (1.47–3.15)**	** < 0.001**	**2.70 (1.94–3.46)**	**0.010**
Differentiation: poor vs. well/moderate	1.55 (1.11–2.16)	0.018		
MVI: yes vs. no	1.22 (0.82–1.83)	0.329		
Satellite: yes vs. no	3.19 (1.81–5.67)	< 0.001		
Steatosis: yes vs. no	0.87 (0.57–1.33)	0.519		
Cirrhosis: yes vs. no	1.34 (0.95–1.90)	0.099		

Independent risk factors for recurrence are indicated in bold

*LLR* laparoscopic liver resection, *BMI* body mass index, *ALBI* albumin–bilirubin, *BCLC* barcelona clinic liver cancer, *PS* posterosuperior segment, *AL* anterolateral segment, *Post-AFP* postoperative α-fetoprotein, *MVI* microvascular invasion

## Discussion

Among all the prognostic risk factors for HCC, recurrence has a significant clinical impact. The operative trends for HCC demonstrate that the proportion of cases performed laparoscopically is increasing [19]. However, in contrast to those of OLR, the patterns of recurrence after LLR for HCC have not been well established. In this study, the majority of patients (78.5%) developed intrahepatic recurrence, and recurrent HCC mainly manifested as multiple lesions, which may reduce the possibility for undergoing radical treatment (liver resection or RFA) after recurrence. Notably, the resection margins were all less than or equal to 1 cm among patients with surgical margin recurrence. However, a wide resection margin and sufficient future liver remnant volume seemed to be paradoxical for the clinical decision. Hence, complete preoperative evaluation and surgical planning by expert surgeons are necessary to achieve an optimal treatment strategy. Regular surveillance by abdominal imaging and tumor markers is appropriate under present recurrence patterns.

The oncology outcomes of LLR compared with OLR for patients with HCC were evaluated using PSM analysis. As shown in Table 2, the patterns, timing, and treatments of recurrence between LLR and OLR were similar. These results suggested that the laparoscopic technique did not change the recurrence of HCC after the operation. On the one hand, although LLR has different intrinsic properties than OLR, en bloc resection and no-touch principles were also followed in endoscopic surgery. On the other hand, laparoscopic surgery is more selective for choosing patients [20]. LLR for proper patients performed by an experienced surgeon can achieve the same results as an open operation [21]. Very little has been found in the literature on the issue of trocar holes and peritoneal metastases after LLR. This study found no statistically significant differences in peritoneal metastases between the two groups. No metastases of the specimen extraction incision or trocar holes were found. Therefore, the correct and rational use of trocar and specimen extraction bags was safe and did not increase the risk of incisional or peritoneal implant metastases in the process of tumor mobilization or extraction. Despite this opinion, extreme caution should be taken to prevent tumor rupture because the risk of peritoneal metastasis of ruptured HCC increases [22]. Therefore, the difference in surgical methods does not affect prognosis.

One of the more noteworthy findings that emerged from this study is that the margin recurrence rate in the NAR group was higher than that in the AR group, although there was no significant difference. There might be several explanations for this finding. First, the small sample size of this study was underpowered to show significant differences between groups. Second, prior studies have shown that the resection method has no impact on the risk of HCC recurrence or survival [23, 24]. Tumor characteristics and biological behaviors could be more crucial for tumor recurrence [25]. Last, NAR at our institution was performed via en bloc resection with a wide surgical margin. Overall, the prognostic impact of the resection margin and resection type has not yet been fully clarified and is an intensely debated topic in the recent liver surgery literature [26–28].

Notably, most (83.3%) patients had recurrence within 2 years after LLR. Patients characterized by a high risk of recurrence could be counseled to receive more intense recurrence surveillance and whether to receive adjuvant therapy. Although no approach has been recommended as a universally accepted adjuvant therapy by current clinical guidelines after curative liver resection, some studies have indicated that prophylactic TACE after curative resection [29, 30] and sorafenib [31] can reduce tumor recurrence and prolong RFS and OS. Thus, predictors from multivariable analysis represent a valuable decision-making tool for clinicians in regards to whether and when to undertake adjuvant therapy.

In reviewing the literature, several oncological features, such as tumor size, tumor number, and MVI, have been identified to be associated with an increased likelihood of HCC recurrence after surgery [3, 32, 33]. It seems that the outcome after recurrent tumor development is already predestined by the characteristics of the original tumor and is out of the control of clinicians. Nevertheless, patients with recurrence who underwent liver resection or RFA had significantly better OS than recurrent patients treated with TACE alone. Choosing the appropriate treatment for recurrent tumors is one approach in which clinicians have the opportunity to influence the outcome.

Poor liver function according to the ALBI grade was shown to be an independent preoperative risk factor for HCC recurrence. The ALBI grade is an objective index of liver reserve function in hepatocellular carcinoma. A large, multi-institutional study validated that the ALBI grade could more accurately predict patient OS [16]. Similarly, another recent study showed that the ALBI grade was strongly associated with the development of tumor recurrence after surgical resection [34]. At present, the Child–Pugh classification is widely used to assess liver function. However, the Child–Pugh score was limited by two highly subjective variables (ascites and encephalopathy). The accurate preoperative identification of patients with poor liver function would greatly help clinicians in selecting appropriate operation procedures (major or minor resection) and follow-up strategies.

It is now well established from a variety of studies that LLR has comparable outcomes to conventional OLR in treating HCC while being less invasive [13, 35]. Compared with conventional open hepatectomy, in LLR, prognostic factors for recurrence differ because of the differentiation in patient selection for surgery and the operating techniques used [36]. Moreover, the results of this study did not show that laparoscopic-related topics, such as liver cirrhosis and the Pringle maneuver, were independent risk factors for HCC recurrence; these topics have also been discussed in previous studies and were shown to have no effect on long-term survival [36, 37]. For some reported risk factors, such as blood loss, postoperative liver failure, and complications, LLR demonstrated superior outcomes. These changes increase the role of patient per se and tumor characteristics in the prediction of recurrence for patients who are treated with LLR. The present study contributes to our understanding of recurrence after LLR for HCC.

Some limitations do exist in our study. First, because of the nature of the retrospective study, all associated bias risks existed. Due to the fact that patients were recruited from a single center, the generalizability of these results is subject to certain limitations. Another limitation of this study was the relatively short follow-up of this cohort of patients. However, to the best of our knowledge, this is the first report concerning the recurrence patterns of LLR for HCC, and the data are from a high-volume HPB center. Therefore, these results are relatively representative and reliable.

## Conclusion

In conclusion, this study provides an accurate and detailed understanding of the patterns and timing of HCC recurrence after LLR. The recurrence patterns suggested that HCC recurrence mainly occurred in the liver and did not increase the risk of incision implantation metastasis. Most cases of recurrence occurred within 2 years after LLR for HCC, suggesting that surveillance should be targeted to early recurrence. Further large, multicenter studies need to be carried out to provide more definitive evidence.

## Supplementary Information

Below is the link to the electronic supplementary material.

Supplementary Figure 1. a. Scatter diagram and b. histograms showing the distributions of propensity scores before and after matching. The treatment unit was the LLR group, and the control unit was the OLR group. (TIF 170 kb)

Supplementary Figure 2. Recurrence-free survival curves between the LLR and OLR groups. (TIF 219 kb)

Supplementary Figure 3. Recurrence-free survival curves between the AR and NAR groups. (TIF 213 kb)

Electronic supplementary material 4 (DOCX 38 kb)
