# Supplementary figures and images for: Patterns, timing, and predictors of recurrence after laparoscopic liver resection for hepatocellular carcinoma: results from a high-volume HPB center
Source: Surg Endosc. 2021 Feb 23;36(2):1215–23. doi: 10.1007/s00464-021-08390-5 (PMC8758625; doi:10.1007/s00464-021-08390-5)

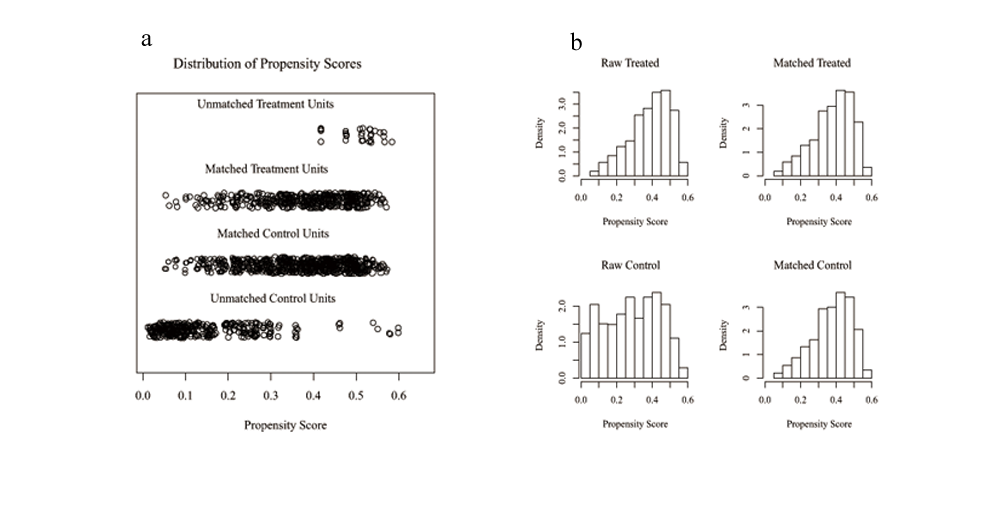

Supplement: Supplementary file 1 — Supplementary Figure 1. a. Scatter diagram and b. histograms showing the distributions of propensity scores before and after matching. The treatment unit was the LLR group, and the control unit was the OLR group. (TIF 170 kb) [file 464_2021_8390_MOESM1_ESM.tif]

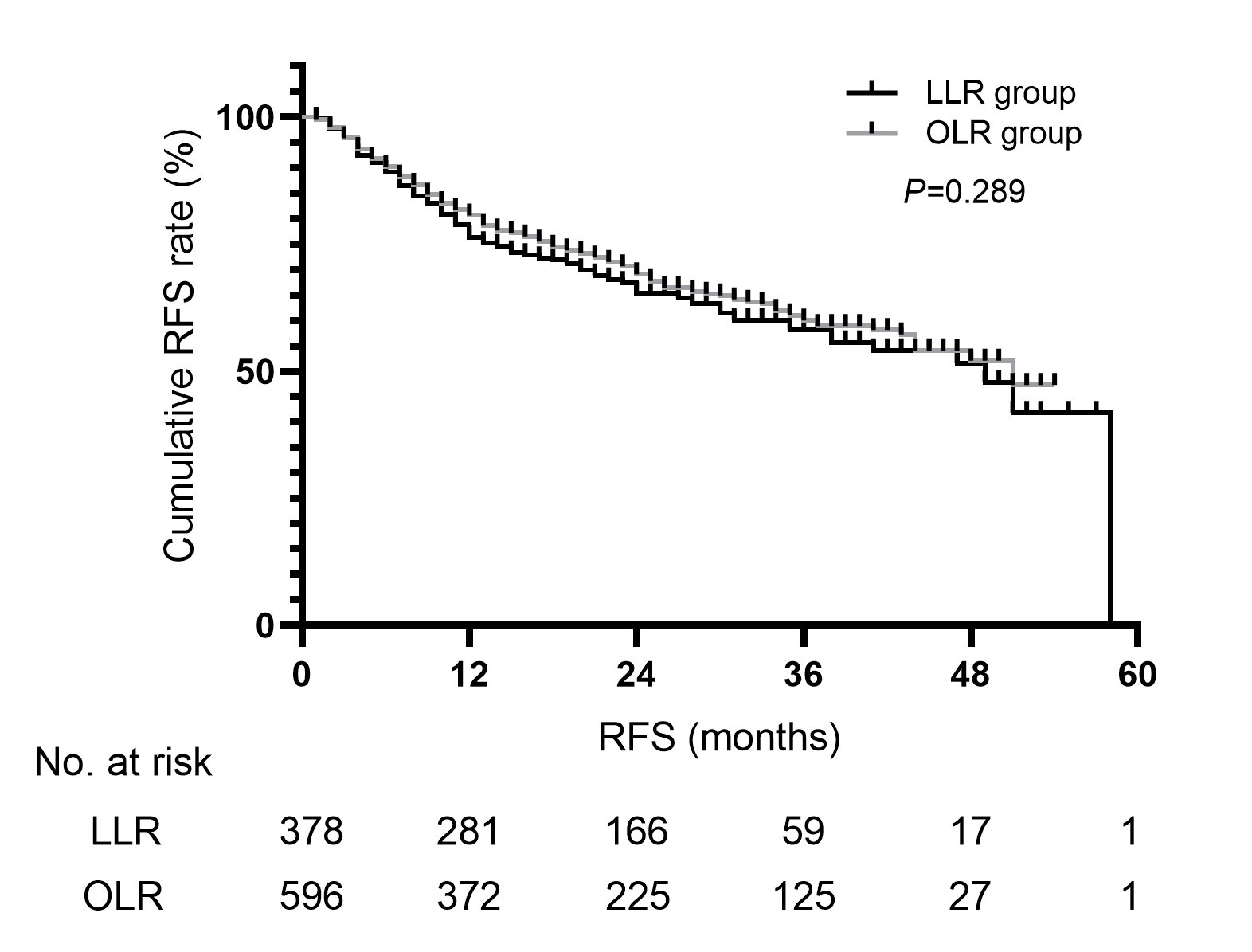

Supplement: Supplementary file 2 — Supplementary Figure 2. Recurrence-free survival curves between the LLR and OLR groups. (TIF 219 kb) [file 464_2021_8390_MOESM2_ESM.tif]

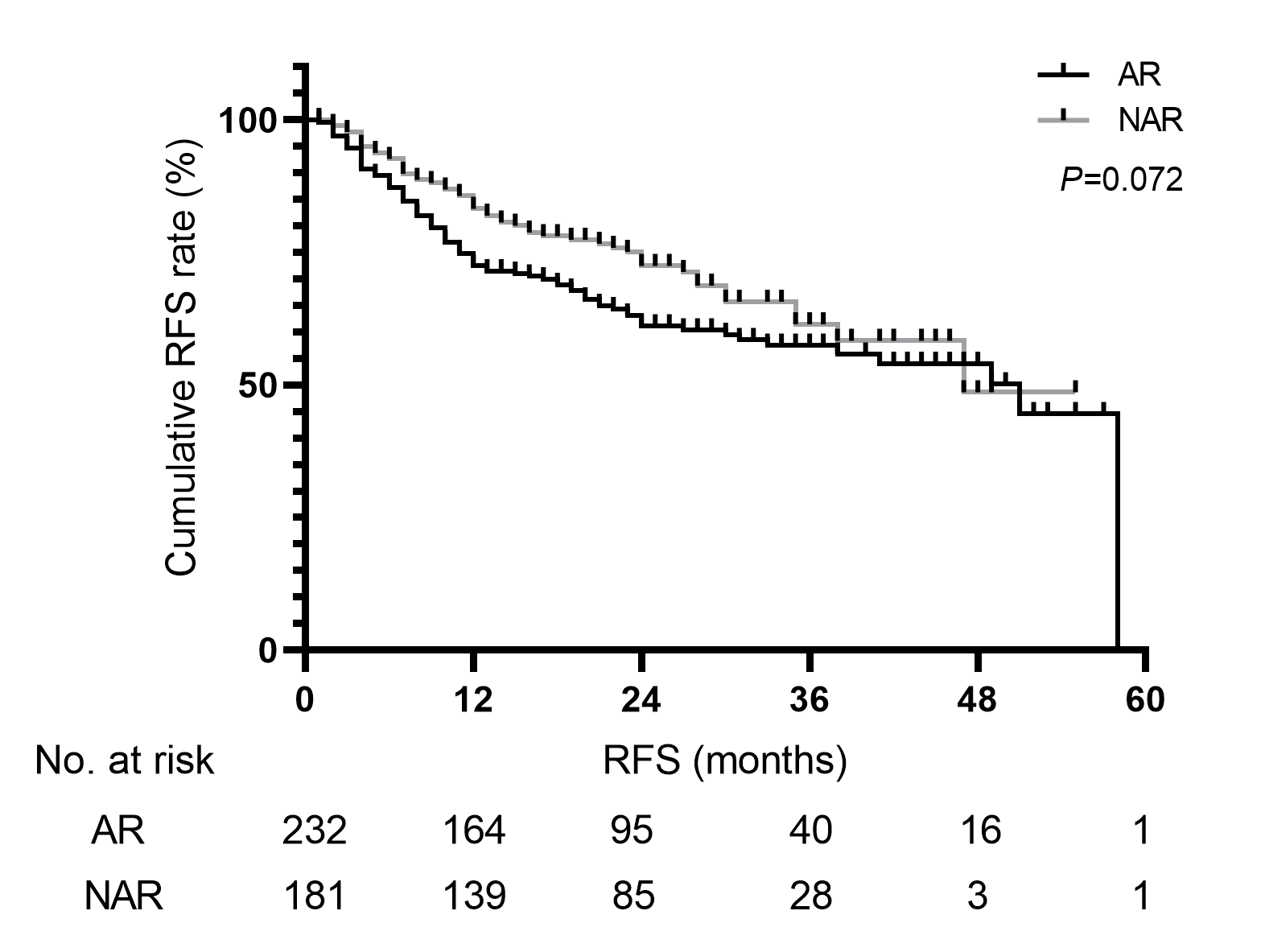

Supplement: Supplementary file 3 — Supplementary Figure 3. Recurrence-free survival curves between the AR and NAR groups. (TIF 213 kb) [file 464_2021_8390_MOESM3_ESM.tif]
