# Supplementary material for: Patterns, timing, and predictors of recurrence after laparoscopic liver resection for hepatocellular carcinoma: results from a high-volume HPB center
Source: Surg Endosc. 2021 Feb 23;36(2):1215–23. doi: 10.1007/s00464-021-08390-5 (PMC8758625; doi:10.1007/s00464-021-08390-5)
Supplement: Supplementary file 4 — Electronic supplementary material 4 (DOCX 38 kb) [file 464_2021_8390_MOESM4_ESM.docx]

| Supplementary Table 1. Demographics, Clinicopathologic, and Treatment Characteristics of Patients before and after PSM | | | | | | |
| --- | --- | --- | --- | --- | --- | --- |
|  | before PSM | | | after PSM | | |
| Variable | LLR (n=425) | OLR (n=902) | *P* value | LLR (n=398) | OLR (n=599) | *P* value |
| Age, mean years (SD) | 53.8 (11.3) | 52.8 (11.5) | 0.015 | 54.4 (11.5) | 53.7 (11.4) | 0.416 |
| Male, n (%) | 357 (84.0) | 759 (84.1) | 0.946 | 333 (83.7) | 497 (83.0) | 0.527 |
| BMI>27, n (%) | 23.6±2.2 | 23.3±3.2 | 0.847 | 23.4±3.0 | 23.3±3.0 | 0.464 |
| ALBI grade, n (%) |  |  | 0.146 |  |  | 0.778 |
| 1 | 344 (80.9) | 692 (76.7) |  | 325 (81.7) | 497 (82.9) |  |
| 2 | 81 (19.1) | 210 (23.3) |  | 73 (18.3) | 102 (17.1) |  |
| BCLC stage, n (%) |  |  | 0.000 |  |  | 0.375 |
| A | 368 (86.8) | 670 (74.3) |  | 341 (85.7) | 506 (84.5) |  |
| B | 47 (11.1) | 124 (13.7) |  | 47 (11.8) | 68 (11.4) |  |
| C | 10 (2.4) | 108 (12) |  | 10 (2.5) | 25 (4.2) |  |
| Etiology |  |  | 0.733 |  |  | 0.842 |
| HBV | 376 (88.5) | 793 (87.9) |  | 351 (88.2) | 525 (87.7) |  |
| HCV | 2 (0.5) | 10 (1.01) |  | 2 (0.5) | 4 (0.6) |  |
| other | 47 (11.1) | 99 (10.9) |  | 45 (11.3) | 69 (11.5) |  |
| HBV-DNA+, n (%) | 245 (57.8) | 525 (58.2) | 0.762 | 216 (54.3) | 347 (57.9) | 0.603 |
| Pre-AFP (ng/ml), median (IQR) | 54.4  (4.8-885.0) | 44.1  (5.3-683.85) | 0.312 | 48  (4.8-697.2) | 51.8  (5.3-808.5) | 0.537 |
| Operation procedure, n (%) |  |  | 0.026 |  |  | 0.002 |
| anatomical resection | 237 (55.8) | 444 (49.2) |  | 221 (55.5) | 273 (45.6) |  |
| non-anatomical resection | 188 (44.2) | 458 (50.8) |  | 177 (44.5) | 326 (54.5) |  |
| Pringle, n (%) | 333 (78.4) | 625 (69.3) | 0.000 | 313 (78.6) | 405 (67.6) | 0.000 |
| CHVIO, n (%) | 19 (4.5) | 130 (14.4) | 0.000 | 18 (4.5) | 80 (13.4) | 0.000 |
| Complications, n (%) |  |  | 0.002 |  |  | 0.000 |
| Clavien-Dindo grade≤II | 405 (95.3) | 793 (87.9) |  | 378 (95.0) | 521 (86.9) |  |
| Clavien-Dindograde≥III | 20 (4.7) | 109 (12.1) |  | 20 (5.0) | 78 (13.1) |  |
| Resection margin, n (%) |  |  | 0.026 |  |  | 0.276 |
| >1cm | 120 (28.2) | 204 (22.6) |  | 112 (28.1) | 150 (25.0) |  |
| ≤1cm | 305 (71.8) | 698 (77.4) |  | 286 (71.9) | 449 (75.0) |  |
| Blood loss (ml), median (IQR) | 200 (70-400) | 200 (150-400) | 0.000 | 200 (100-400) | 200 (100-400) | 0.036 |
| Tumor location, n (%) |  |  | 0.000 |  |  | 0.000 |
| right/left liver | 16 (3.8) | 151 (16.7) |  | 16 (4.0) | 36 (6.0) |  |
| right anterior section | 17 (4.0) | 72 (8.0) |  | 17 (4.3) | 45 (7.5) |  |
| right posterior section | 20 (4.7) | 86 (9.5) |  | 20 (5.0) | 46 (7.7) |  |
| posterosuperior segment | 96 (22.6) | 235 (26.1) |  | 94 (23.6) | 189 (31.6) |  |
| anterolateral segment | 276 (64.9) | 358 (39.7) |  | 251 (63.1) | 283 (47.2) |  |
| Tumor size (cm), median (IQR) | 3.5 (2.5-5.0) | 5.5 (3.2-9.0) | 0.000 | 4.0 (3.0-5.0) | 4.0 (2.8-6.0) | 0.090 |
| Tumor number, n (%) |  |  | 0.333 |  |  | 0.615 |
| single | 354 (83.3) | 732 (81.2) |  | 328 (82.4) | 501 (83.6) |  |
| multiple | 71 (16.7) | 170 (18.8) |  | 70 (17.6) | 98 (16.4) |  |
| Tumor differentiation, n (%) |  |  | 0.000 |  |  | 0.219 |
| well- moderate | 264 (62.1) | 450 (50.1) |  | 240 (60.3) | 336 (56.4) |  |
| poor | 161 (37.9) | 449 (49.9) |  | 158 (39.7) | 260 (43.6) |  |
| Microvascular invasion, n (%) | 87 (20.5) | 295 (32.7) | 0.000 | 85 (21.4) | 141 (23.5) | 0.420 |
| Satellite nodules, n (%) | 21 (4.9) | 135 (15.0) | 0.000 | 20 (5.0) | 57 (9.5) | 0.009 |
| Cirrhosis, n (%) | 260 (61.2) | 486 (53.9) | 0.012 | 237 (59.5) | 344 (57.4) | 0.506 |
| BMI, body mass index; ALBI, albumin-bilirubin; BCLC, Barcelona Clinic Liver Cancer; HBV: hepatitis B virus; HCV: hepatitis C virus; Pre-AFP, preoperative α-fetoprotein; CHVIO, continuous hemi-hepatic vascular inflow occlusion; SD, standard deviation; IQR, interquartile range | | | | | | |

|  | | | | | | | | | | | | | | | | | | | | |  | |  |  |  |  |
| --- | --- | --- | --- | --- | --- | --- | --- | --- | --- | --- | --- | --- | --- | --- | --- | --- | --- | --- | --- | --- | --- | --- | --- | --- | --- | --- |
| Supplementary Table 2. Subgroup analysis of recurrence patterns and timing by risks factors | | | | | | | | | | | | | | | | | | | | | | | | | |  |
|  | ALBI | | |  | Surgical margin | | |  | Postoperative AFP | | |  | Tumor size | | |  | Tumor number | | |  | | Tumor location | | | |  |
| Variable | grade 1 (n=344) | grade 2 (n=81) | *P* value |  | >1 cm (n=120) | ≤1 cm (n=305) | *P* value |  | ≤8 ng/ml (n=267) | >8 ng/ml (n=158) | *P* value |  | <5 cm (n=288) | ≥5 cm (n=137) | *P* value |  | single (n=354) | multiple (n=71) | *P* value |  | | anterolateral (n=276) | | posterosuperior (n=96) | *P* value |  |
| Number of recurrences, n (%) | | 112 (32.6) | 32 (39.5) | 0.235 |  | 31 (25.8) | 113 (37.0) | 0.049 |  | 61 (22.8) | 83 (52.5) | 0.000 |  | 76 (26.4) | 68 (49.6) | 0.000 |  | 109 (30.8) | 35 (49.3) | 0.003 |  | | 95 (34.4) | | 27 (28.1) | 0.258 |
| Patterns, n (%)^a^ | |  |  |  |  |  |  |  |  |  |  |  |  |  |  |  |  |  |  |  |  | |  | |  |  |
| liver only | | 75 (66.9) | 24 (75.0) | 0.387 |  | 17 (54.8) | 82 (72.6) | 0.084 |  | 39 (63.9) | 60 (72.3) | 0.285 |  | 56 (73.7) | 43 (63.3) | 0.177 |  | 75 (68.8) | 24 (68.6) | 0.979 |  | | 64 (67.4) | | 17 (63.0) | 0.671 |
| margin | | 12 (10.7) | 3 (9.4) | 0.827 |  | 3 (9.7) | 12 (10.6) | 0.827 |  | 8 (13.1) | 7 (8.4) | 0.364 |  | 8 (10.5) | 7 (10.3) | 0.964 |  | 10 (9.2) | 5 (14.3) | 0.389 |  | | 10 (10.5) | | 5 (18.5) | 0.285 |
| liver & distant | | 8 (7.1) | 2 (6.3) | 0.861 |  | 3 (9.7) | 7 (6.2) | 0.540 |  | 4 (6.6) | 6 (7.2) | 0.876 |  | 1 (1.3) | 9 (13.2) | 0.013 |  | 5 (4.6) | 5 (14.3) | 0.050 |  | | 7 (7.4) | | 2 (7.4) | 0.995 |
| distant | | 10 (9.0) | 2 (6.3) | 0.629 |  | 5 (16.1) | 7 (6.2) | 0.091 |  | 5 (8.2) | 7 (7.4) | 0.959 |  | 6 (7.9) | 6 (8.8) | 0.840 |  | 11 (10.1) | 1 (2.8) | 0.319 |  | | 8 (8.4) | | 2 (7.4) | 0.864 |
| peritoneal | | 7 (6.3) | 1 (3.0) | 0.496 |  | 3 (9.7) | 5 (4.4) | 0.527 |  | 5 (8.2) | 3 (3.6) | 0.236 |  | 5 (6.6) | 3 (4.4) | 0.571 |  | 8 (7.3) | 0 | 0.221 |  | | 6 (3.7) | | 1 (6.3) | 0.589 |
| RFS, median mo. (range) | | 10 (2-58) | 10 (1-49) | 0.801 |  | 13 (1-47) | 10 (2-58) | 0.979 |  | 11 (2-58) | 8 (1-51) | 0.302 |  | 11 (2-58) | 8 (1-51) | 0.138 |  | 10 (1-58) | 10 (2-49) | 0.759 |  | | 10 (2-58) | | 8 (1-47) | 0.125 |
| a. percentage of the number of recurrences  ALBI, albumin-bilirubin; AFP, α-fetoprotein; RFS, recurrence-free survival | | | | | | | | | | | | | | | | | | | | | | | | | |  |
